# Association between prostate specific antigen levels and coronary artery angioplasty

**DOI:** 10.15171/jrip.2017.26

**Published:** 2016-12-15

**Authors:** Arezoo Khosravi, Eghlim Nemati, Mahdi Soleimanian, Neda Raesi, Shahin Abbaszadeh

**Affiliations:** ^1^Atherosclrosis Research Center, Baqiyatallah University of Medical Sciences, Tehran, Iran; ^2^Nephrology and Urology Research Center, Baqiyatallah University of Medical Sciences, Tehran, Iran

**Keywords:** Acute coronary syndromes, Percutaneous coronary intervention, Prostate specific antigen, Stable ischemic heart disease

## Abstract

**Introduction:** Prostate-specific antigen (PSA) is a protein, whose serum levels changes during various physiologic and pathologic situations. Recently, the relationship between PSA and cardiologic disorders has been assessed.

**Objectives:** The purpose of this study was to assess the association of percutaneous coronary intervention (PCI) complications with PSA serum levels.

**Patients and Methods:** In this study, 100 eligible patients undergoing PCI were included. The total PSA serum values were analyzed pre- and post-procedure. The association between PSA levels with age, gender, inflammatory (C-reactive protein [CRP] and white blood cell [WBC]), cardiogenic (troponin, CK-MB, echocardiography and angiography results), and nephrology (creatinine) properties was investigated.

**Results:** Changes in the level of PSA pre- and post-PCI was not significant (P=0.2). However, based on the pathology, patients with acute coronary syndrome (ACS) had a significant difference in the levels of PSA compared to cases of stable ischemic heart disease (SIHD) (*P*=0.008). Moreover, the effect of gender on the changes in PSA level following PCI was conclusive. There was no association between the direct effect of PCI parameters or PCI complications on PSA level changes.

**Conclusion:** The results showed that PSA levels were affected by the etiology of cardiac disorders instead of therapeutic methods like PCI.

**Implication for health policy/practice/research/medical education:**

In an investigation, total PSA serum values before and after the procedure were examined. We observed that differences in the levels of PSA before and after PCI was not significant. However, based on the pathology, patients with ACS had a significant discrepancy in the level of PSA compared to cases of SIHD.

## Introduction

Percutaneous coronary intervention (PCI) is a technique for treating coronary heart disease. In 2008 more than one million people underwent PCI in the United States, and this rate is expected to be increased by 1%-5% in the next decade (1).

Not being an exception from other invasive procedures, PCI has complications classified as major and minor. Stroke, myocardial infarction (MI) and death are major, while transient ischemia, contrast nephropathy and vascular events are minor complications.

There are some uncommon complications such as coronary dissection, stent thrombosis and no-reflow phenomenon (2-4).

Despite large studies and improvements in PCI and its complications, physicians are still very concerned about PCI’s adverse effects.

Recently, studies have reported that prostate-specific antigen (PSA) is related to cardiovascular diseases (5-7). Despite its name, various studies have revealed that PSA is also expressed outside of prostate glands such as female tissue (including breast, ovarian, and endometrial tissues) and body fluids (amniotic fluid, milk, and breast cyst fluids). Investigations show that MI, prolonged cardiopulmonary resuscitation (CPR), cardiogenic shock, bypass of coronary vessels during cardiac surgeries, reduced peripheral vascular elasticity due to hypertension and cardiac stenting can increase PSA levels. Also, a recent study by Durmaz et al in Turkey, concluded that, there may be an association between serum PSA and free PSA levels and diagnosis of acute coronary syndrome (ACS) and extent of coronary artery disease (CAD) (7).

## Objectives

We hypothesized that serum PSA changes may be correlated with PCI cardiovascular complications. We conducted this study to evaluate whether we can predict the risk of cardiovascular event after PCI.

## Patients and Methods

This study was a cohort study conducted at Baqiyatallah University Hospital, Tehran, Iran from June 2013 to December 2013. Around 100 patients between 40 to 60 years admitted to cardiac wards for PCI were included in this study.

Exclusion criteria were presence of malignancy, benign prostatic hyperplasia, a recent history of prostate biopsy, inflammatory disease, and a history of lower urinary tract symptoms, surgery or trauma within the previous month, CPR or cardiogenic shock on admission, urinary retention, and catheterization before and after PCI.

Information regarding age, gender, body mass index (BMI) were recorded. Also, a 10 cc blood sample was collected from the upper limb veins of each subject 24 hours before the PCI procedure and another sample was collected the next day postoperative. The angiographic video files of the patients were thereby reviewed to determine the kind and severity of the lesions and the number of attacked vessels. All the included patients underwent echocardiography before PCI and some data like ejection fraction (EF), the mitral regurgitation (MR), and cardiac wall motion abnormalities were collected. We also recorded changes in the electrocardiogram (ECG) such as T inversion, depression or elevation of ST-segment and arrhythmias for each participant. The blood samples were assessed for CK-MB, troponin I and PSA (free and total) using chemo-immune laboratory tests and for C-reactive protein (CRP) using a photometric method with Biosystem kit. The results of PCI, its probable complications such as dissection, rupture of the vessels and the remaining of occlusion were also recorded in the patients’ files.

Patients were visited before discharge and assessed for chest pain, dyspnea and ECG. One month after the PCI procedure, phone calls were made to the subjects asking them about the remission or recurrence of the symptoms, re-hospitalization because of cardiac problems and probable secondary angiography.

### Ethical issues

Written informed consent was obtained from each patient after a detailed explanation of the objectives, purpose of the study and the protocol of the study which adhered to the ethical principles stated in the “Declaration of Helsinki” and was approved by the Baqiyatallah University of Medical Sciences ethics committee (Ethical number #867).

### Statistical analysis

The data was analyzed by SPSS version 20 (SPSS, Chicago, IL, USA). The quantitative variables were presented as the mean ± standard deviation (SD), and the qualitative variables were presented in units of frequency and percentage. Comparison of the means between groups was made using the parametric or non-parametric test according to one sample Kolmogorov-Smirnov test result for normal distribution. The comparison between qualitative variables was made using the chi-square test. To assess the relationship between the various variables and the laboratory data, regression analysis was used. *P* values less than 0.05 were considered statistically significant.

## Results

A total of 100 patients were included in this study, of whom 45% were women, with a mean age of 54.8 ± 7.3 years, a mean BMI of 26.3 ± 11.2 kg/m^2^ and a mean clearance of creatinine 1.1 mg/dL.

Forty-one percent of the participants exhibited documented history of type two diabetes, 50% with hypertension, 17% with dyslipidemia, 9% with previous MI, 14% with the previous PCI, 3% with prior coronary artery bypass grafting (CABG) and 3% with chronic renal failure.

Among our participants, 32 cases were admitted to the hospital because of ACS and 68 cases of stable ischemic heart disease (SIHD).

Aspirin was the most common therapeutic agent (62%) in the drug history of patients.

Of all the participants, 28 had EF of more than 55%, 52 had EF of 45%-54%, 14 patients had EF of 30%-44%, and others had lower EF levels.

Regional wall motion abnormalities (RWMA) were recorded in 11 patients, and seven subjects had levels of MR.

Electrocardiography of the patients was evaluated for probable electrical heart disorders. The results showed that most patients had normal sinus rhythm ECG. T wave inversion was the most impaired ECG pattern (20%) and the least observed impaired ECG was left bundle branch block (LBBB) (2%).

Angiographic videos revealed that 55% of the subjects had single vessel disease (SVD), 34% had two-vessel disease (2VD), and 11 patients had three vessel disease (3VD).

A majority (85%) of the participants underwent implantation with drug-eluting stents (DES), and the bare metal stents (BMS) were utilized for the rest.

In nine patients complications accompanied the PCI procedure. Moreover, there was chest pain after PCI in 35 patients. Among these 35 patients six had ECG changes and two patients needed emergency PCI because of stent thrombosis. Three patients required re-hospitalization during the first month after PCI.

Table 1 depicts the analysis of inflammatory parameters and PSA before and after PCI in the participants.

**Table 1:** Inflammatory parameters and PSA before and after PCI

**Parameters**	**Before PCI**		**After PCI**		***P*** ** value**
	**Mean**	**SD**	**Mean**	**SD**	
WBC (cells/mL)	7159.1	2479.7	6841.7	2857.4	0.52
Creatinine (mg/dL)	1.14	0.72	1.24	0.82	0.07
CRP (mg/L)	6.00	7.31	6.06	3.64	0.97
Troponin I (ng/mL)	0.88	2.65	0.67	2.21	0.29
CK-MB (U/L)	11.21	25.37	9.13	15.10	0.35
Total PSA (ng/mL)	1.48	0.76	1.79	1.49	0.20
Free PSA (ng/mL)	0.208	0.095	0.147	0.061	0.18

Abbreviations: PSA, Prostate-specific antigen; PCI, percutaneous coronary intervention; CRP, C-reactive protein; WBC, white blood cell.

To assess the relationship between the age of the participants and the changes in total and free PSA, we applied the Pearson’s correlation analysis which showed no significant relationship among them (r = -0.12, *P* = 0.67 for total PSA and r = 0.11, *P* = 0.92 for free PSA).

Furthermore, a significant difference in serum total and free PSA levels between patients with RWMA versus patients without this problem was detected (*P* = 0.04).

The mean of the changes in the total PSA level before and after PCI in women and men were 0.036±0.001 ng/mL and 0.09 ± 0.03 ng/mL, respectively which was significantly different (*P* = 0.037). There were no significant changes in free PSA between genders before and after PCI (*P* = 0.17).

The changes in total PSA in ACS patients (0.87 ± 0.28) was significantly different from SIHD patients (0.39 ± 0.26 ng/mL; *P* = 0.008).

Data analysis also revealed that the proportion of attacked vessels in angiography (*P* = 0.32), the insertion of the stent during PCI (*P* = 0.59) and the type of stent (*P* = 0.14) were not related to PSA levels.

Also, the total and free PSA levels had no role in after-PCI complications (*P* = 0.22 for total PSA and *P* = 0.17 for free PSA) and in re-hospitalization after PCI (*P* = 0.82 for total PSA and *P* = 0.13 for free PSA).

The relationship between the changes in other blood factors and PSA levels are shown in Table 2.

**Table 2:** Relationship between blood inflammatory factor levels and PSA levels

		**CK-MB changes (U/L)**	**Troponin I changes (ng/mL)**	**CRP changes (mg/L)**	**Creatinine changes (mg/dL)**	**WBC level changes (cells/mL)**
PSA changes (ng/mL)	Pearson correlation	0.431	0.564	-0.347	-1.000^**^	1.000^**^
	*P* value	0.569	0.436	0.653	0.43	0.24.
Free PSA changes (ng/mL)	Pearson correlation	-0.024	-0.040	0.014	0.024	0.034
	*P* value	0.891	0.819	0.939	0.908	0.856

Abbreviations: PSA, Prostate-specific antigen; CRP, C-reactive protein; WBC, white blood cell.

## Discussion

PSA is a 33 kDa glycoprotein that has been known as a member of the human kallikrein family (hK3) of serine proteases (8,9). Despite its name, it is not specified for prostatic diseases, semen and male gender (10). Different studies have reported its relation with cardiovascular diseases (5,7).Prolonged CPR, cardiogenic shock after acute MI, bypass of coronary vessels during cardiac surgeries and reduced peripheral vascular elasticity due to hypertension are all proposed to increase PSA levels (5,6,11).

Previous studies have propounded that steroid hormones affect the PSA levels and the reduction in sex hormones by aging, and menopause in women, lead to lower PSA plasma measures (12,13).According to this point, we can justify the differences in PSA values among both genders in our study.

Our results showed a significant difference of change in serum PSA between ACS and SIHD patients. Nevertheless, no remarkable increase in the PSA level after conducting PCI in patients suffering from cardiac problems was detected which could be due to low sample size in our study. This result apparently stated that the elevation in free and total PSA was mostly because of inflammatory and acute reactions. It is clear that inflammatory markers have a significant role in the detection of unstable lesions. We hypothesized that serum PSA level, such as other acute phase reactants, could predict the risk of ACS patients and also may assist in the diagnosis and treatment of unstable patients. Also, significant difference in serum total and free PSA levels between patients with and without RWMA increases the probability of our hypothesis because RWMA occurs in acute injury and represents high-risk disease. However, other studies are needed to evaluate and confirm this hypothesis.

Taussig et al, for the first time proposed the theory that after cross-clumping abdominal aorta during aortocoronary bypass surgery, a relative infarction in prostate will happen and that was due to the pelvic ischemia which made for the increase in PSA levels (14).This theory was put to experiment by serial checking of PSA after surgery (15,16).Koller-Strametz et al reported the increase in PSA, 12-24 hours post-CPR and the resulting decrease starting seven days after the procedure (16). Parlaktas and colleagues also observed a significant difference in PSA levels between the first and the fifth days after bypass surgery. One can assume that the main reason for PSA elevation after the surgery is revascularization which is stronger the first days and after that subsides (17).Some researchers believe that urethral catheterization and slight trauma to the prostate can elevate serum PSA levels (18,19). Taking into account, this effect and adjusting it in further studies, there was still a relationship between cardiac diseases and PSA values. Hypovolemic shock and ACS are believed to increase PSA levels independently (20).In a recent another study, Ozcan et al concluded that coronary artery stenting is associated with a rise in serum total and free PSA values 24 hours after the intervention and these elevated levels return to baseline by 30 days after the procedure (21). Ozcan et al proved that neither coronary artery stent implantation nor coronary angiography affected free/total PSA ratio. They concluded that this ratio could be used as a marker for prostate malignancy during the first 30 days after coronary artery stenting.

However, there are reports of diminution of PSA in some patients during acute MI. Patanè et al, concluded that when the elevation of PSA occurred during acute MI, coronary lesions were frequent and often more severe in comparison with the time when diminution of PSA took place (22-24).

To put all these and our study results, in a nutshell, the changes in total PSA (not free PSA) can give us a pattern of underlying cardiac disease or its risk factors. We also showed that stenting had no significant effect on PSA. Moreover, the findings showed no significant relationship between the on-site and post PCI complications and PSA levels.

We came to the point that the activity of the kallikrein–kinin system was linked to inflammation. Patanè et al believed that the elevation in free and total PSA was mostly because of inflammatory reactions and also because of the changes in their precursor amounts, pro-PSA (24). Pro-PSA is converted to active PSA by human kallikrein 2 (hK2) (9,25,26).It has been reported that hK2 might not alone be able to activate pro-PSA in vitro and there are also other proteases involved in this event. Cleaving high-molecular-weight kininogen, hK2 produces bradykinin. It also activates the single-chain urokinase type plasminogen activator (uPA) (24,26).The researchers suggested that the levels of PSA were more reliable if interpreted in combination with CRP values. Nevertheless, despite the difference in serum PSA change before and after PCI between ACS and SIHD patients, no direct relation between these two inflammatory markers (PSA and CRP) before and after PCI was detected. In addition, our study showed no relationship between other inflammatory, cardiac or renal markers and PSA. However, our study is the only one paying attention to this aspect and other studies with bigger sample sizes in future are warranted.

## Conclusion

The current study showed no remarkable increase in the PSA level after conducting PCI in patients suffering from cardiac problems in general. Dividing the patients into two groups of ACS and SIHD, the difference appears significant. We also detected no significant association between the on-site and post PCI complications and PSA values. Moreover, our study showed no relationship between other inflammatory, heart or kidney markers and PSA. However, our study can be a trigger for other investigators to work on PSA as a predictor of ACS and its complications.

## Limitations of the study

According to our knowledge, our research is the only one paying attention to this purpose, and other large sample size studies are required in future when indicated.

## Authors’ contribution

AK and MS participated in all experiments, coordinated the data-analysis and contributed to the writing of the manuscript. MS coordinated the acquisition of data. AK designed the research plan and organized the study. MS performed analysis and interpretation of data. NR and EN prepared the final manuscript.

## Ethical considerations

Ethical issues (including plagiarism, data fabrication, double publication) have been completely observed by the authors.

## Conflict of interests

The authors declare no conflict of interest.

## Funding/Support

This article is extracted from cardiology thesis of Mehdi Soleimanian. This study was supported by a grant from Baqiyatallah University of Medical Sciences (Grant # 867, 2013).
